# Emotional distress, occupational stress and burnout among Family Doctors in Europe: Monitoring and testing of interventions is required

**DOI:** 10.1080/13814788.2021.1985998

**Published:** 2021-10-11

**Authors:** Andree Rochfort, Claire Collins, Jako Burgers

**Affiliations:** a Irish College of General Practitioners, Dublin, Ireland; b Dutch College of General Practitioners, Utrecht, The Netherlands

**Keywords:** General practice/family medicine, stress, burnout, emotional distress, intervention

## Abstract

In recent years, the medical literature from a wide range of medical specialities has exploded with publications on the theme of emotional distress, stress and burnout in the practice of medicine. Improving the work-life of health care providers is necessary to optimise health system performance. COVID-19 has caused considerable additional pressure on health services across Europe and there have been calls for interventions to address the psychological and occupational stress caused by the pandemic. Although there is an ongoing need to monitor these factors among family physicians, and other staff working in primary care across Europe, we must also identify supports and promote them. Further research is needed to explore causative factors and provide convincing evidence in relation to effective interventions.

KEY MESSAGES

- There is a need to promote doctors’ wellbeing in order to maintain patient safety and high-quality healthcare and to optimise health system performance.
- It is time to move beyond descriptive research to provide evidence on effective interventions.

The pandemic caused by COVID-19 has brought out a particular phenomenon and highlighted it to the entire world. Medical doctors are also human.

Doctors can catch coronavirus infections at work and die. Doctors can catch milder versions of coronavirus and survive. Like patients, medical doctors get cancer, heart attacks, thrombosis and osteoporosis. Medical doctors too, can suffer from anxiety, depression, psychosis and dementia. Medical doctors can experience profound bereavement, exquisite joy, guilt, shame, stress and burnout. Doctors can also be patients.

Many people’s jobs are associated with high levels of responsibility. People who work as engineers, air traffic controllers and pilots make decisions at critical moments while at work. One could argue the same point for many other employees and employers. The key difference between the medical job and other responsible jobs is the high level of personal accountability and potential for public scrutiny of decisions and outcomes. Under pressure of a new consultation every few minutes, complex clinical decisions can be challenged and scrutinised by others with the wisdom of hindsight, at a leisurely pace, sometimes years after they have occurred. Critical decision making is the hallmark of medical work.

COVID-19 has caused unprecedented pressure on health services and health workers across Europe, on a scale not seen outside major wars. There have been calls for interventions to address the impact of the psychological effects and occupational stress caused by the pandemic [1,2]. Although this is not limited to the medical professions [1], frontline healthcare workers have been shown to have high levels of anxiety, depression and acute stress disorder, which may adversely impact their own well-being and patient safety [2].

However, concern regarding emotional distress, stress and burnout among health professionals and the impact on quality of care is not new. An International Health Policy Survey of primary care physicians in 11 countries showed that perceived job stress had increased in all countries from 2015 to 2019 [3]. As far back as the 1980s, the relationship between perceived stress levels, GP satisfaction and quality of care was reported [4]. Burnout is shown to be associated with lower patient satisfaction and reduced patient health outcomes [5].

Before the COVID pandemic, many countries had shortages of health workers, including in family medicine. Emerging data suggest that mental health difficulties, perceived stress and burnout could be further exacerbated by the ongoing COVID-19 pandemic. An inadequate response to support family doctors experiencing work-related stress and burnout will lead to a more pronounced manpower problem if family physicians need to stop working or decide to leave the profession altogether. The potential future impact on clinicians is likely to negatively impact overall healthcare delivery.

Healthcare leaders and managers responsible for quality and safety of healthcare delivery need to develop and test interventions – individual, practice-based, community and national strategies – to limit and manage the impacts of psychosocial occupational hazards on both health professionals and their patients. Terminology can sometimes be prohibitive to developing evidence-based targeted solutions as terms such as burnout and stress are sometimes used interchangeably and there is a need for clarity regarding the definition of concepts used in international studies.

Research documenting and monitoring emotional distress, occupational stress and burnout is important but we should also encourage research, which explores the root causative factors and exacerbating factors and that provides convincing evidence in relation to effective interventions. In fact, if one searches the topic areas of published general practice/family medicine papers over the past 10 years, a high proportion refers to burnout but most simply document its existence; some of these papers discuss predictors but few have tested interventional strategies.

We believe that interventions for measuring changes in structures or processes of care delivery should be designed to the highest level of quality for valid outcome evaluation. Hence, this type of research needs to identify both the specific predisposing and trigger factors as well as the environmental contexts which influence interventions and outcomes. Family practice operates as a complex work environment, and there are a multitude of confounding factors that can affect the choice of intervention, such as workload volume, time pressure, complexity of consultations, consultation style, risk of litigation, night and weekend emergency work, payment systems, options for local community referral, waiting times for specialist opinions, access to diagnostics, IT system functionality, health literacy, patient expectations and adherence to the European Working Time Directive. Healthcare services are labour intensive; however, the staffing levels and workplace environments of family practice also vary considerably within countries and between countries.

We recognise and agree with the urgent need to evaluate and monitor emotional distress, occupational stress and burnout among family physicians across Europe and identify related supports and promote them. However, to advance the evidence base and begin to consider what solutions might work best for different people in the various settings and health care systems, general practice/family medicine research must move beyond descriptive research and begin to investigate the adaptation and implementation of solutions.
